# Carbamazepine Induces Platelet Apoptosis and Thrombocytopenia Through Protein Kinase A

**DOI:** 10.3389/fphar.2021.749930

**Published:** 2021-09-23

**Authors:** Weiling Xiao, Kangxi Zhou, Mengnan Yang, Chenglin Sun, Lan Dai, Jian Gu, Rong Yan, Kesheng Dai

**Affiliations:** ^1^ Jiangsu Institute of Hematology, Cyrus Tang Medical Institute, The First Affiliated Hospital and Collaborative Innovation Center of Hematology, State Key Laboratory of Radiation Medicine and Protection, Medical College, Soochow University, Key Laboratory of Thrombosis and Hemostasis, Ministry of Health, Suzhou, China; ^2^ Department of Immunology, School of Basic Medical Sciences, Weifang Medical University, Weifang, China; ^3^ Department of Hematology, Northern Jiangsu People’s Hospital, Yangzhou, China

**Keywords:** carbamazepine, thrombocytopenia, PKA, platelet apoptosis, PDE3A

## Abstract

Carbamazepine is extensively used worldwide to treat a wide range of disorders such as epilepsy, peripheral neuralgia and bipolar disorder. Thrombocytopenia and hemorrhage have been identified in multiple carbamazepine-treated patients. However, the underlying mechanism remains poorly understood. Here, we show that platelets undergo apoptosis after carbamazepine treatment. The apoptotic platelets induced by carbamazepine are rapidly removed *in vivo*, which accounts for thrombocytopenia. We found that carbamazepine treatment attenuates the phosphorylation level of bcl-xl/bcl-2-associated death promoter (BAD), vasodilator-associated stimulated phosphoprotein (VASP) and GPIbβ in platelets, indicating an inhibition effect on protein kinase A (PKA). We further demonstrated that carbamazepine reduced PKA activity through PI3K/Akt/PDE3A signaling pathway. Pharmacological activation of PKA or inhibition of PI3K/Akt/PDE3A protects platelets from apoptosis induced by carbamazepine. Importantly, PDE3A inhibitors or PKA activator ameliorates carbamazepine-mediated thrombocytopenia *in vivo*. These findings shed light on a possible mechanism of carbamazepine-induced thrombocytopenia, designating PDE3A/PKA as a potential therapeutic target in the treatment of carbamazepine-induced thrombocytopenia.

## Introduction

Epilepsy, one of the most common neurological disorders, affects about 70 million people around the world (Hirtz et al., 2007; Cagnetti et al., 2014). Antiepileptic drugs (AEDs) such as phenytoin, carbamazepine (CBZ) and valproate, are key drugs for the treatment of epilepsy (Perucca and Tomson, 2011; Perucca et al., 2018). Despite the development of new AEDs, CBZ is still widely recognized as a first-line monotherapy for newly diagnosed subjects with focal epilepsy (Glauser et al., 2013; Nolan et al., 2015; Zhu et al., 2015). In addition to epilepsy, CBZ is widely used in a multiple other neurological and psychiatric disorders (Lima et al., 2002; Bialer, 2012; Fricke-Galindo et al., 2018). However, prolonged CBZ therapy is frequently associated with severe hematological alterations, including agranulocytosis (Kumari et al., 2011), thrombocytopenia (Ishikita et al., 1999) and even aplastic anemia (Tohen et al., 1995; Blackburn et al., 1998). Among these alterations, it should be noted that CBZ-induced isolated thrombocytopenia has been reported in clinical application (Kimura et al., 1995; Ishikita et al., 1999; Finsterer et al., 2001). And thrombocytopenia induced by CBZ, which can cause life-threatening hemorrhage, frequently results in great risk of discontinuation during routine treatment. Though immunological factors and pharmacology of individual drugs are the proposed mechanisms, the exact underlying mechanism for CBZ-induced thrombocytopenia has not yet been established.

Platelets, the small anucleate cytoplasmic fragments released from megakaryocytes, are the second most abundant cell in the circulation. There are approximately one trillion platelets circulating in the blood of an adult human, and because the lifespan of an individual platelet is only 8–10 days, 100 billion new platelets must be produced daily from bone marrow megakaryocytes in order to maintain normal platelet counts (Kaushansky, 2006). Senescent platelets undergo apoptosis before being removed in the liver and spleen (Kaplan and Saba, 1978; Deppermann et al., 2020). The steady-state number of mature platelets is the result of the balance between platelet production and destruction, which is strictly regulated under physiological conditions. Thrombocytopenia is caused either by increased breakdown or by ineffective production of platelets. The impaired platelet formation mediated by chemical factors is usually accompanied by myelosuppression, which demonstrates as cytopenias in all three peripheral blood lineage -- anaemia, leucopenia and thrombocytopenia (Egorin et al., 1984; Tsuji et al., 2017). Selective peripheral cytopenia is frequently caused by shortening blood cell survival rather than suppressing cell production. Moreover, accumulating evidence indicates that accelerated platelet apoptosis can be triggered by many pathological stimuli (Yeh et al., 2010; Gafter-Gvili et al., 2011; Tang et al., 2014) or chemical compounds (Mason et al., 2007), leading to thrombocytopenia.

Platelet apoptosis is a mitochondria-mediated intrinsic form of programmed cell death, which is initiated by the members of BCL-2 family, leading to mitochondrial transmembrane potential depolarization and caspases activation (Zhao et al., 2017). It has been shown that BAD Ser155 can be phosphorylated by PKA, thereby enhancing its binding to 14-3-3 and promoting cell survival (Datta et al., 2000; Lizcano et al., 2000). Dephosphorylated BAD Ser155 mediated by PKA inhibition disassociates from 14-3-3, followed by combination with Bcl-xL to form a heterodimer on mitochondria. And then proapoptotic BAX is released leading to apoptosis (Yang et al., 1995). PKA is highly expressed in platelets and its activity is strictly regulated. We recently reported that PKA determines platelet life span and survival by regulating apoptosis. PKA regulates platelet apoptosis through mediating BAD Ser155 phosphorylation. Reduced PKA activity incurred by various stimuli accelerates intrinsic programmed platelet apoptosis *in vitro* and rapid platelet clearance *in vivo* (Zhao et al., 2017). It has been found that PI3K/Akt signaling pathway may be activated by CBZ, which plays important roles in nucleated cells (Kawaguchi et al., 2013; Elsherbiny et al., 2019). And our group recently demonstrated that Akt locates the upstream of PKA to regulate platelet apoptosis (Chen et al., 2018). Therefore, we speculate that PKA-mediated platelet apoptosis might be involved in the pathogenesis of CBZ-induced thrombocytopenia.

To clarify the molecular mechanism of CBZ-induced isolated thrombocytopenia, we investigated the biological role of CBZ in platelet apoptosis. We found that CBZ dose-dependently induced platelet apoptosis through inhibiting PKA activity. Moreover, we elucidated that PI3K/Akt/PDE3A signaling pathway serves as the upstream mediator of PKA inhibition in CBZ-induced platelet apoptosis. Calpain inhibition mediated by CBZ counteracts the role of activated Akt, in which prevents platelets from being hypersensitive. Inhibition or genetic ablation of PI3K/Akt can only slightly rescue the platelets from clearance. However, activation of PKA can robustly protect platelets from apoptosis and clearance induced by CBZ. Thus, our findings reveal the pathogenesis of thrombocytopenia in CBZ treated individuals and, more importantly, suggest therapeutic strategies for thrombocytopenia caused by CBZ application.

## Materials and Methods

### Mice

*Bad* ^*−/−*^ mice were generated on a 129/SvJ background as described previously (Ranger et al., 2003). *Akt* ^*−/−*^ mice were generated on 129/Ola and C57BL/6 mixed background (Yang et al., 2003). All mutant mice had been backcrossed onto the C57BL/6J background for at least 10 times to generate syngeneic gene-deficient mice. C57BL/6J WT mice were purchased from JOINN Laboratories. Balanced groups of male and female mice aged 8–12 weeks and weighing 20–25 g were used for experiments. All mice were housed in a specific pathogen-free facility in the Laboratory Animal Center of Soochow University in accordance with the National Institute of Health Guide for Care and Use of Laboratory Animals. During our research, we followed the standard biosafety procedures of Soochow University. All animal experiments were approved by the Ethics Committee of the First Affiliated Hospital of Soochow University (2017.065).

### Reagents and Materials

JC-1 (C2005) and Forskolin (1,612) were purchased from Beyotime Institute of Biotechnology (Beyotime, Shanghai, China). FITC-conjugated lactadherin (BLAC-FITC) was purchased from Haematologic Technologies (Essex Junction, VT, United States). Antibodies against caspase-3 (9,662), *β*-actin (4,979), phospho-Akt Ser 473 (4,060), phospho-Akt Thr 308 (13,038), Akt (9,272), Bcl-xL (2,764), Bax (2,772), phospho-p53 ser15 (9,284), phospho-VASP (Ser 157) (84,519) and GAPDH (5,174) were from Cell Signaling Technology (Beverly, MA, United States). A23187 (sc-3591), antibodies against Bak (sc-832), phospho-BAD Ser 155 (sc-101641) and Filamin A (sc-17749) were from Santa Cruz Biotechnology (Santa Cruz, CA, United States). FITC-conjugated anti-human P-selectin antibody (304,904), PE-conjugated anti-mouse CD41 antibody (133,906) and FITC-conjugated anti-CD41 antibody (ab19708) were purchased from Biolegend (San Diego, CA, United States). FITC-conjugated PAC-1 (340,507) was purchased from BD Biosciences (San Jose, CA, United States). Anti-phospho GPIbβ (Ser166) polyclonal antibody was kindly provided by Professor Xiaoping Du (University of Illinois, Chicago, IL, United States). LY294002 (M1925) and Wortmannin (M2053) were purchased from Abmole Bioscience (Houston, TX, United States). Cilostazol (S1294) and carbamazepine (S1693) were purchased from Selleck (Houston, TX, United States). AKT inhibitor Ⅷ (AKTI Ⅷ, HY-10355), MK 2206 (HY10358) and Milirone (HY-14252) were purchased from MedChem Express (Princeton, NJ, United States). 8-Br-cAMP (B7880) and ADP (A2754) were obtained from Sigma (St Louis, MO, United States). U46619 (538,944) was purchased from Calbiochem (La Jolla, CA, United States). Thrombin (#386) and collagen (#385) were from Chrono-log Corp (Havertown, PA, United States).

### Blood Collection and Platelet Isolation

Platelet and blood cell counts were performed with Sysmex XP-100 Hematologic Analyzer (Sysmex Corporation, Kobe, Japan). Informed consent was obtained from all subjects, and the human studies were approved by the Ethics Committee of the First Affiliated Hospital of Soochow University. The platelets from health volunteers were prepared as previously described (Zhao et al., 2017; Chen et al., 2018). Briefly, whole blood of healthy controls aged 22–50 years was drawn from the elbow vein and anticoagulated with 1/7 volume of acid-citrate-dextrose (ACD, 2.5% trisodium citrate, 2.0% D-glucose, 1.5% citric acid). Platelet-rich plasma (PRP) was collected from the whole blood by 200 g centrifugation for 11 min. Platelets were washed with CGS buffer (0.123 M NaCl, 0.033 M D-glucose, 0.013 M trisodium citrate, pH 6.5), resuspended in modified Tyrode’s buffer (MTB) (2.5 mM Hepes, 150 mM NaCl, 2.5 mM KCl, 12 mM NaHCO_3_, 5.5 mM D-glucose, 1 mM CaCl_2_, 1 mM MgCl_2_, pH 7.4) to a final concentration of 3 × 10^8^/mL. Then, washed platelets were incubated at 22°C for 1–2 h prior to application.

For the preparation of murine platelets, whole blood from mice was obtained from postorbital vein using 1/7 volume of ACD as anticoagulant. Platelets were washed twice with CGS buffer and resuspended in MTB to a concentration of 3 × 10^8^/mL and allowed to be incubated at 22°C for 1–2 H.

### *In vitro* CBZ Treated Platelet Assays

Human washed platelets were incubated with CBZ in a dose-dependent manner at 37°C for 70 H. In order to avoid lesion caused by extrusion and contamination, platelets were kept gently stirring under a sterile condition. For antiepilepsy purpose, it is the only method for CBZ to achieve the therapeutic dose range (4–12 μg/ml) in patients under steady-state conditions (Hill et al., 2019). However, the peak serum concentration of CBZ can achieve more than 36 μg/ml (Brahmi et al., 2006; Yang et al., 2018). The concentration of 12 μg/ml is approximately equivalent to 50 μM. Relative longer time of incubation and higher concentrations were selected to compensate shear stress free *in vitro*. Murine washed platelets were treated with 100 μM CBZ at 37°C for 6–8 H. The decreased incubation period in murine platelets is due to its hypersensitivity for CBZ and shorten life span. In inhibition experiments, human platelets were preincubated with diverse inhibitors or their vehicle control at 37°C for 10 min, and then treated with CBZ at 37°C for 70 H.

### Flow Cytometry

Platelet apoptosis was detected by mitochondrial transmembrane potential (Δψ_m_) depolarization or phosphotidylserine (PS) exposure. Δψ_m_ depolarization in platelets was detected by JC-1, and depolarization was characterized as the decrease in JC-1 aggregates. PS exposure was detected by FITC-labeled lactadherin. Platelet activation was detected by P-selectin (CD62-P) expression and integrin αⅡbβ3 activation. P-selectin expression was analyzed with FITC-labeled anti-CD62P antibody. Activated GPⅡb/Ⅲa was detected by FITC-labeled PAC-1 binding platelets. Platelets were analyzed by flow cytometer (FC 500, Beckman-Coulter).

### Western Blot

Washed platelets (3 × 10^8^/mL) were incubated with indicated concentrations of CBZ or pre-incubated with corresponding inhibitors at 37°C for 10 min before CBZ application, and then lysed with 1/4 volume of 5 × lysis buffer on ice for 30 min and boiled at 95°C for 10 min. Proteins were separated by sodium dodecyl sulfate polyacrylamide gel electrophoresis (SDS-PAGE). After blocking, membranes were incubated with primary antibodies and HRP-conjugated secondary antibodies. Blots were developed by ECL chemiluminescence system. Quantification was performed with ImageJ software.

### CBZ-Induced Platelet Clearance *in vivo*

C57BL/6 mice or transgenic mice (8–10 weeks) received 1.2 mg/kg CBZ or vehicle control in 100 μL sterile phosphate-buffered saline (PBS) through tail vein. The whole blood, anti-coagulated with 3.8% trisodium citrate, was collected from the post-glomus venous plexus. Platelet counts was measured by Sysmex XP-100 Hematologic Analyzer. For PKA activated experiments, C57BL/6 mice were intravenously injected with a single dose of PKA activator 8-Br-cAMP (2.5 mg/kg) or vehicle control. Ten minutes after 8-Br-cAMP injection, the mice were injected with 1.2 mg/kg CBZ through the tail vein.

### Post Transfusion Experiment

Washed murine platelets labeled with 5 μg/mL calcein were incubated with 100 μM CBZ at 37°C for 2 H and injected intravenously into acceptor mice (1 × 10^8^ platelets in 100 μL MTB). The blood collected from the post-glomus venous plexus at 1 (baseline), 15 and 30 min after transfusion. The whole blood was labeled with PE-conjugated anti-CD41 antibody. The percentage of calcein-labeled platelets remaining in circulation was assessed by flow cytometry. For mutant platelet clearance, C57BL/6J mice were *i.v.* transfused with calcein-labeled CBZ-treated platelets from transgenic mice or WT littermates. For inhibition of PDE3A activity, calcein-labeled platelets were pretreated with milrinone (10 μM), cilostazol (10 μM) or vehicle control at 37°C for 10 min before incubation with 100 μM CBZ *in vitro*.

### Platelet Aggregation

Platelet aggregation was recorded in a Chrono-Log-Model 700 aggregometer (Havertown, PA, United States) at a stirring speed of 1,200 rpm at 37°C. Platelet-rich plasma or washed human platelets (3 × 10^8^/mL) were pretreated with 100 μM CBZ at 37°C for 5 min. The pretreated PRP or washed platelets were stimulated with indicated concentrations of different agonists. Platelet aggregation was monitored continuously over 10 min.

### Statistical Analyses

All data were expressed as mean ± standard deviation (SD) of at least three independent experiments. Statistical analysis was performed using GraphPad Prism six software. Numeric data were evaluated by one-way (for single variant) or two-way (for multiple variants) ANOVA analysis of variance. Two groups were analyzed by the two-tailed Student’s *t*-test. For all analyses, a *p* value < 0.05 was considered to indicate statistical significance.

## Results

### CBZ Induces Platelet Apoptosis *in vitro* and Apoptosis-dependent Thrombocytopenia *in vivo*

**FIGURE 1:**
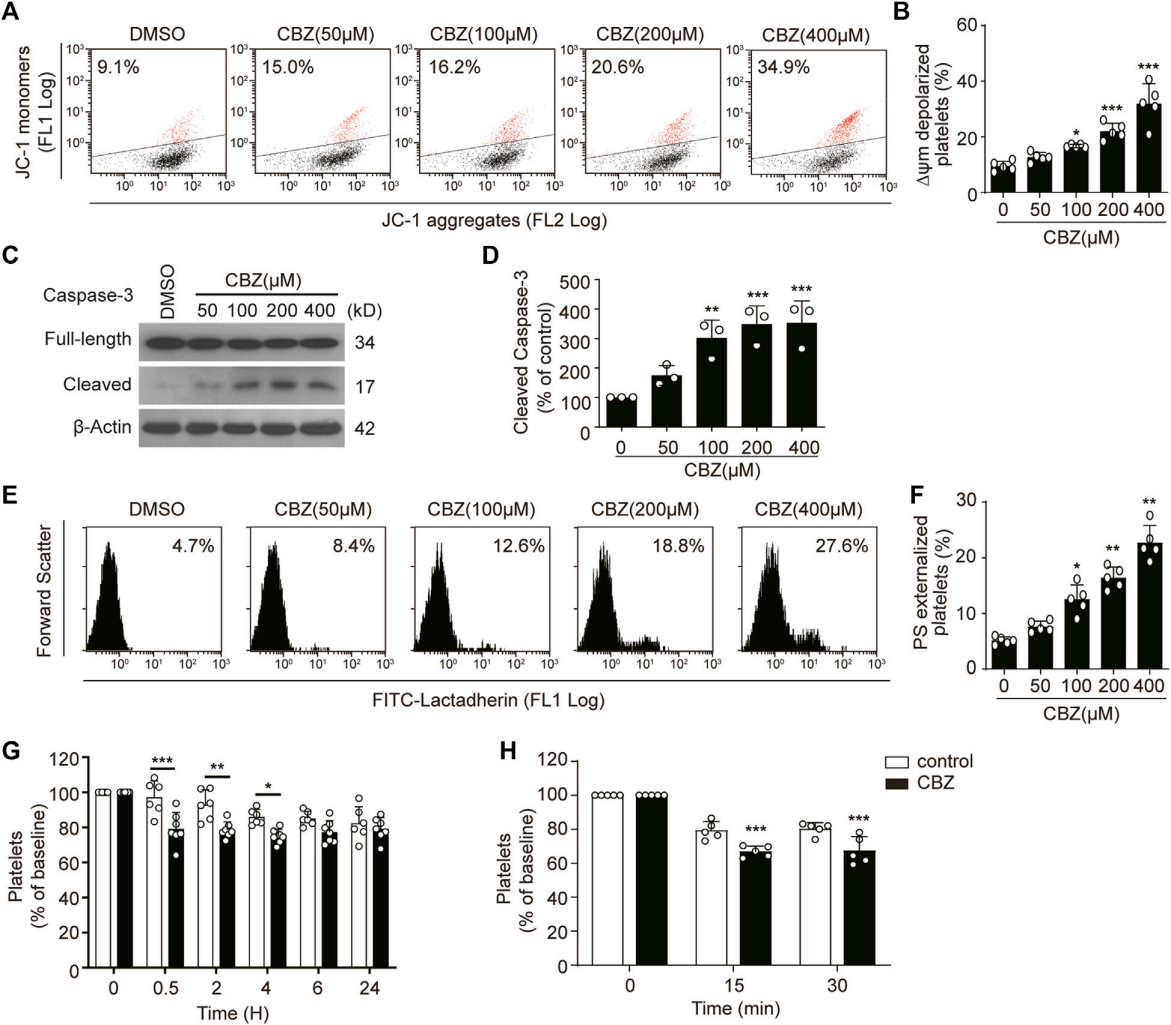
CBZ induces platelet apoptosis *in vitro* and apoptosis-dependent thrombocytopenia *in vivo*. **(A–F)** Human washed platelets were incubated with indicated concentrations of CBZ or vehicle control at 37°C for 70 H. **(A,B)** Representative flow cytometric figures **(A)** and quantification **(B)** of platelet △ψ_m_ depolarization; *n* = 5 **(C,D)** Caspase-3 was analyzed with anti-caspase-3 antibody by Western blot **(C)**. Densitometry of immunoblot for cleaved caspase-3 from the Western blot data; *n* = 3 **(D)**. **(E,F)** Representative flow cytometric figures **(E)** and quantification **(F)** of PS exposure of platelets; *n* = 5. **(G)** C57BL/6J mice were *i.v*. injected with 1.2 mg/kg CBZ or vehicle control and then platelet counts were determined at the indicated time points; *n* = 6 for control, *n* = 7 for CBZ treatment group. Baseline is defined as the platelet number before CBZ injection **(H)** Calcein-labeled mouse platelets were incubated with 100 μM CBZ or vehicle control at 37°C for 2 H and then were transfused into WT mice; *n* = 5 for each group. Baseline is the percentage of calcein positive platelets immediately after platelet infusion. Data are expressed as mean ± SD; *p < 0.05, **p < 0.01, ***p < 0.001 versus control group, one-way ANOVA in **(A–F)** and two-way ANOVA prior to Bonferroni *post hoc* test in **(G,H)**.

To investigate the pathogenesis of CBZ-induced isolated thrombocytopenia, we examined the biological role of CBZ in platelet apoptosis, since apoptosis determines the lifespan of platelets (Zhao et al., 2017; Wei and Harper, 2019). We found that CBZ dose-dependently induced Δψ_m_ depolarization in platelet (Figures 1A,B). In addition, CBZ activates caspase-3 in a dose-dependent manner, as shown by the increased presence of 17-kDa fragments (Figures 1C,D). Caspases can bind with other apoptogenic enzymes to disrupt plasma membrane integrity, driving PS externalization during apoptosis (Schoenwaelder et al., 2009; Leytin, 2012). We found that CBZ indeed incurred PS exposure dose-dependently (Figures 1E,F).

Apoptotic platelets can be removed by macrophages in the liver. Thus, we reasoned that CBZ may induce thrombocytopenia through shortening platelet life span *in vivo*. Therefore, we next examined the role of platelet apoptosis in CBZ-induced thrombocytopenia and platelet clearance. In order to illustrate the direct effect of CBZ on platelet counts, a single dose of CBZ was inject into murine blood through the tail vein. After intravenously injection, the circulating platelets showed a decline trend from 0.5 h in WT mice, and murine platelet counts began to recover after 4 h and returned to normal level at about 6 h after injection (Figure 1G). Moreover, to eliminate the influence of allergic immune factors on thrombocytopenia, platelet transfusion experiment was employed to detect the effect of CBZ on platelet clearance *in vivo*. We found that platelets pretreated with CBZ *in vitro* result in more rapid clearance compared with that in vehicle group (Figure 1H). Taken together, these data demonstrate that CBZ directly elicits intrinsic programmed apoptosis, which leads to apoptosis-dependent thrombocytopenia *in vivo*.

### CBZ Reduces Platelet PKA Activity

**FIGURE 2:**
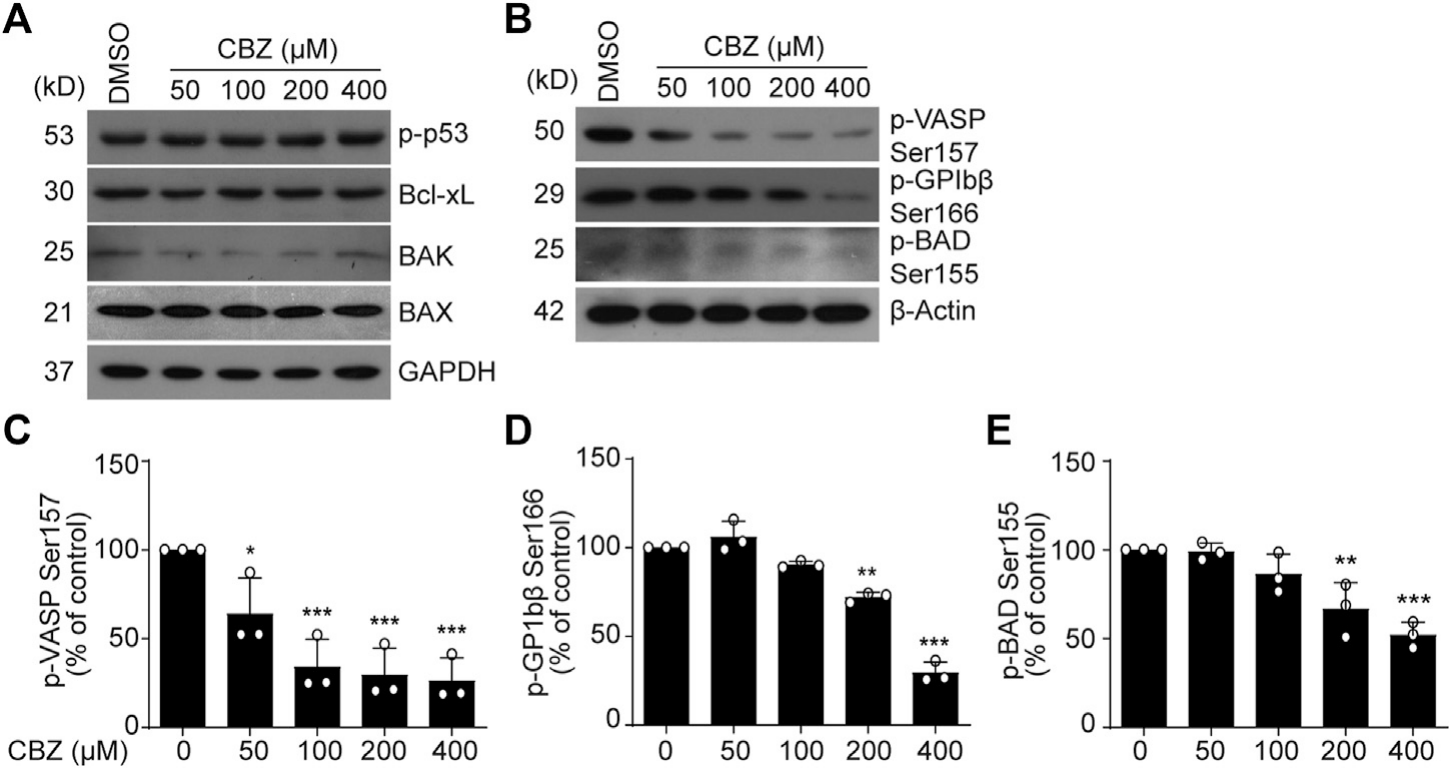
PKA activity is reduced in CBZ treated platelet. **(A,B)** Washed human platelets were incubated with indicated concentrations of CBZ or vehicle control at 37°C for 70 H. Indicated proteins were analyzed with various primary antibodies by Western blot in human platelets. Data are representative of three separate experiments. **(C–E)** Quantification of phosphorylated VASP Ser157 PS **(C)**, GPIbβ Ser166 **(D)** and BAD Ser155 **(E)**; *n* = 3. *p < 0.05, **p < 0.01, ***p < 0.001 compared with control by one-way ANOVA.

Next, we investigated the mechanism of CBZ-induced platelet apoptosis. First, we tested apoptotic proteins in the CBZ-treated platelets. We found that p-p53, Bcl-xL, Bak and Bax remain unchanged in the CBZ-treated platelets (Figure 2A). PKA activity reduction should result in dephosphorylation of BAD at Ser155, which incurs platelet apoptosis (Zhao et al., 2017). Therefore, we detected PKA activity and phosphorylation of BAD at Ser155. We found that PKA activity in platelets was dose-dependently reduced with increasing concentrations of CBZ (Figures 2B–E), as indicated by dephosphorylation of the PKA substrates VASP (Figures 2B,C) (Smolenski et al., 1998) and GPIbβ Ser166 (Figures 2B,D) (Wardell et al., 1989). Phosphorylation of BAD at Ser155, which is regulated by PKA (Lizcano et al., 2000), was also reduced by CBZ in a dose dependent manner (Figures 2B,E). These data indicate that PKA activity is reduced in platelets with CBZ application.

### PKA Inhibition Is Responsible for CBZ-Induced Platelet Apoptosis

**FIGURE 3:**
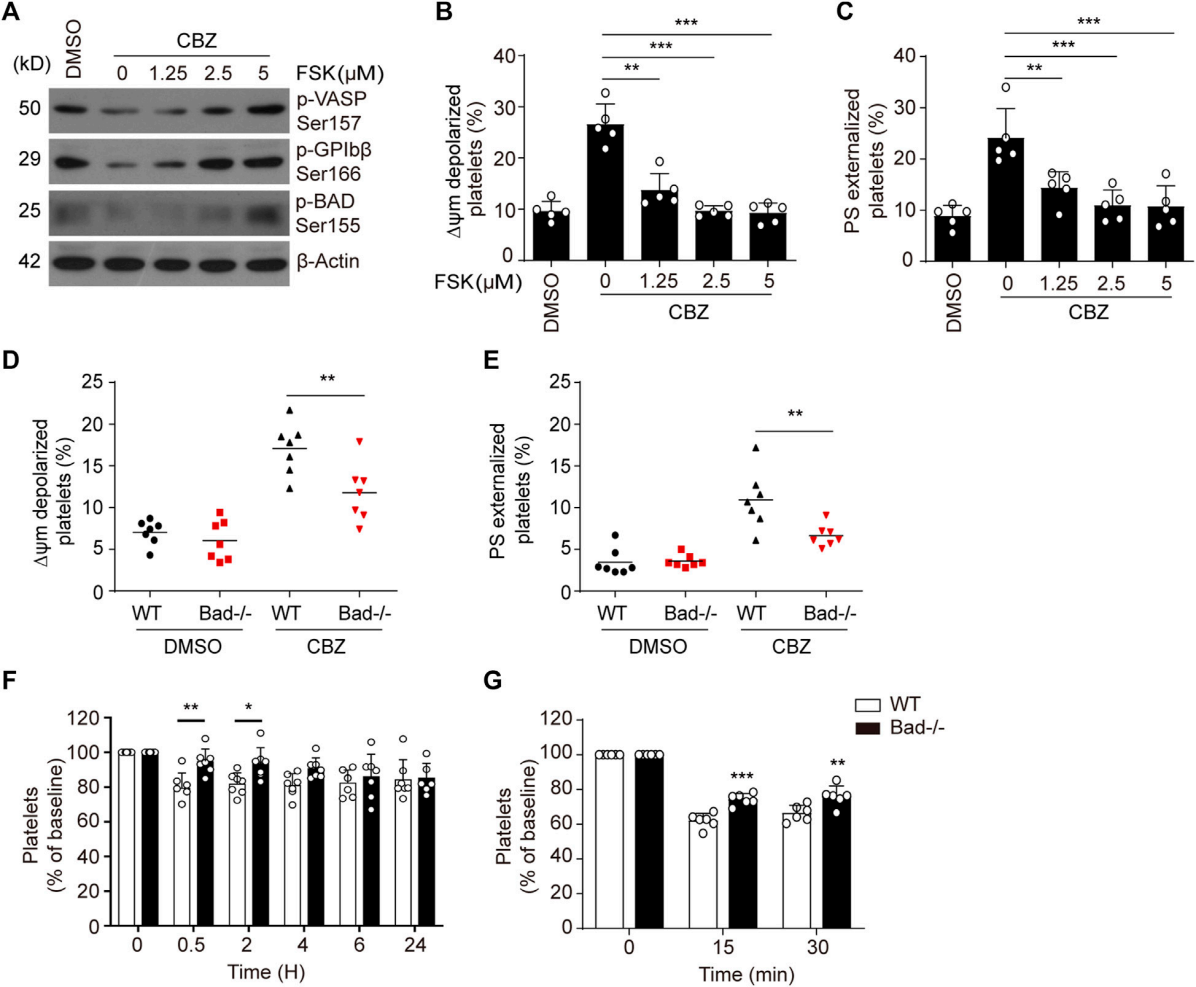
Activation of PKA rescues platelets from apoptosis induced by CBZ. **(A–C)** Washed human platelets were pre-treated with indicated concentrations of PKA activator forskolin or vehicle control at 37°C for 5 min followed by 100 μM CBZ incubation at 37°C for 70 H. **(A)** Western blot analysis of phosphorylated VASP at Ser157, phosphorylated GPIbβ at Ser166, and phosphorylated BAD at Ser155 in platelets. (B and C) Platelet △ψ_m_ depolarization **(B)** and PS exposure **(C)** were detected by flow cytometry; *n* = 5. Data are expressed as mean ± SD. **p < 0.01, ***p < 0.001, compared with control with one-way ANOVA. **(D,E)** Washed platelets from WT or *Bad* ^*−/−*^ mice were incubated with 100 μM CBZ at 37°C for 6 H. Platelet △ψ_m_ depolarization **(D)** and PS exposure **(E)** were detected by flow cytometry; *n* = 7 for each group. Data are expressed as mean ± SD. Horizontal lines are the representatives of median values and each dot indicates one mouse. **p < 0.01, ***p < 0.001, compared with WT control by Student’s *t*-test. **(F)** WT and *Bad* ^*−/−*^ mice were *i.v.* injected with a single dose of CBZ (1.2 mg/kg). After injection, platelet counts were determined at the indicated time points; *n* = 7 for each group. **(G)** C57BL/6J mice were *i.v.* transfused with calcein-labeled CBZ-treated platelets from *Bad* ^*−/−*^ or WT littermates; *n* = 6 for each group. **(F,G)** Data are mean ± SD; *p < 0.05, **p < 0.01, ***p < 0.001 versus control group, two-way ANOVA prior to Bonferroni *post hoc* test.

We previously reported that PKA determines the life span of platelets by regulating apoptosis (Zhao et al., 2017; Chen et al., 2018). To further elucidate the link between CBZ-mediated platelet apoptosis and PKA inhibition, we tested whether PKA activation may affect platelet apoptosis. Forskolin, a well-known PKA activator, was utilized to selectively elevate PKA activity. As we expected, forskolin dose-dependently reversed CBZ-induced PKA inhibition, as indicated by improved phosphorylation of VASP at Ser157, GPIbβ at Ser166 and BAD at Ser155 (Figure 3A). Evaluation of ΔΨ_m_ depolarization and PS exposure revealed that CBZ-induced apoptotic events were dose-dependent reduced by forskolin (Figure 3B,C). Given that PKA inhibition mediates the dephosphorylation of BAD at Ser155 to carry out platelet apoptosis, we speculated that BAD knockout may reverse CBZ-induced platelet apoptosis. To test this, we employed BAD deficient platelets, which lack PKA substrate, and found that compared with WT platelets, CBZ-induced apoptotic events were markedly reduced in *Bad* ^*−/−*^ platelets (Figure 3D,E).

Platelet apoptosis may lead to platelet clearance *in vivo* (Quach et al., 2018). We therefore investigated the *in vivo* influence of BAD on CBZ-induced platelet clearance. Compared with WT mice, *Bad* ^*−/−*^ mice displayed less thrombocytopenia after CBZ injection (Figure 3F). Meanwhile, BAD absence resulted in obviously decreased platelet clearance induced by CBZ (Figure 3G). Collectively, these data indicate that PKA is likely the dominant target of CBZ-mediated platelet apoptosis, and also suggest that PKA activation has potential prevention and therapeutic value for CBZ mediated thrombocytopenia.

### PI3K/Akt/PDE3A Signaling Pathway Plays an Essential Role in CBZ-Reduced Human Platelet PKA Activity

**FIGURE 4:**
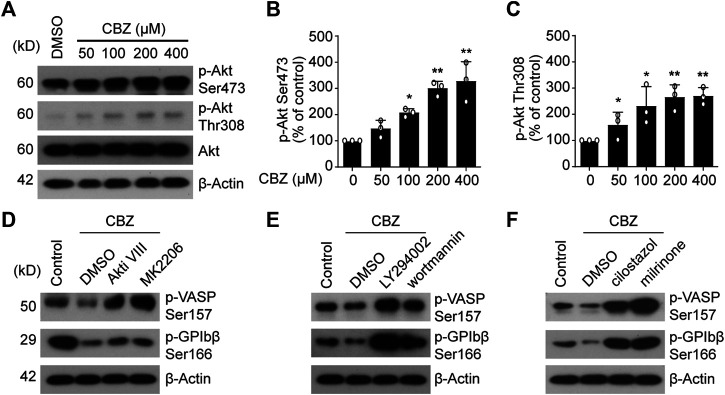
PI3K/Akt/PDE3A signaling pathway plays an essential role in CBZ-reduced PKA activity. **(A)** The expression of *p*-Akt Ser-473 and Thr-308 were detected by Western blot analysis from human platelets incubated with CBZ or vehicle at 37°C for 8 H; **(B,C)** Histogram showing the relative normalized expression for each phosphorylated protein; *n* = 3. *p < 0.05, **p < 0.01, compared with control, one-way ANOVA. **(D–E)** Washed human platelets were pretreated with Akt inhibitors **(D)** MK2206 (6 μM) and Akti Ⅷ (2 μM), PI3K inhibitors **(E)** LY294002 (20 μM) and wortmannin (100 nM), PDE3A inhibitors **(F)** milrinone (10 μM) and cilostazol (10 μM), or vehicle control at 37°C for 10 min followed by 100 μM CBZ application at 37°C for 16 H. Phosphorylation of VASP and GPIbβ was analyzed by Western blot analysis; data are representative of three separate experiments.

Then we explored the reason of PKA inhibition caused by CBZ. It was recently reported that PI3K/Akt may be activated by CBZ to undergo critical roles in eukaryocytes (Elsherbiny et al., 2019; Kawaguchi et al., 2013). Therefore, it is possible that PI3K-Akt may be activated by CBZ in platelets. In support of this, we found that CBZ activates Akt in a dose-dependent manner (Figures 4A–C). Our group previously reported that phosphodiesterase (PDE3A) that lies downstream of PI3K-AKT, hydrolyzes intracellular cAMP leading to decreased PKA activity (Chen et al., 2018). In view of this, we speculate that PKA inhibition mediated by CBZ should be realized by activating PI3K/Akt/PDE3A signal pathway. Therefore, selective inhibition of this pathway maybe reverse the downregulated PKA activity induced by CBZ. To test this, human platelets were pretreated with PI3K, Akt or PDE3A inhibitors followed by CBZ administration. The results show that pretreatment with inhibitors of PI3K (LY294002 and wortmannin), Akt (MK 2206 and Akti Ⅷ) or PDE3A (milrinone and cilostazol) markedly reverses CBZ-mediated platelet PKA inhibition (Figures 4D–F), as indicated by the elevated phosphorylation of VASP at Ser157 and GPIbβ at Ser166. These data demonstrate that PI3K/Akt/PDE3A signaling pathway is the upstream of PKA in CBZ-treated platelets.

### Selective Inhibition of PI3K/Akt/PDE3A Pathway Protects Platelets From CBZ-Induced Apoptosis

**FIGURE 5:**
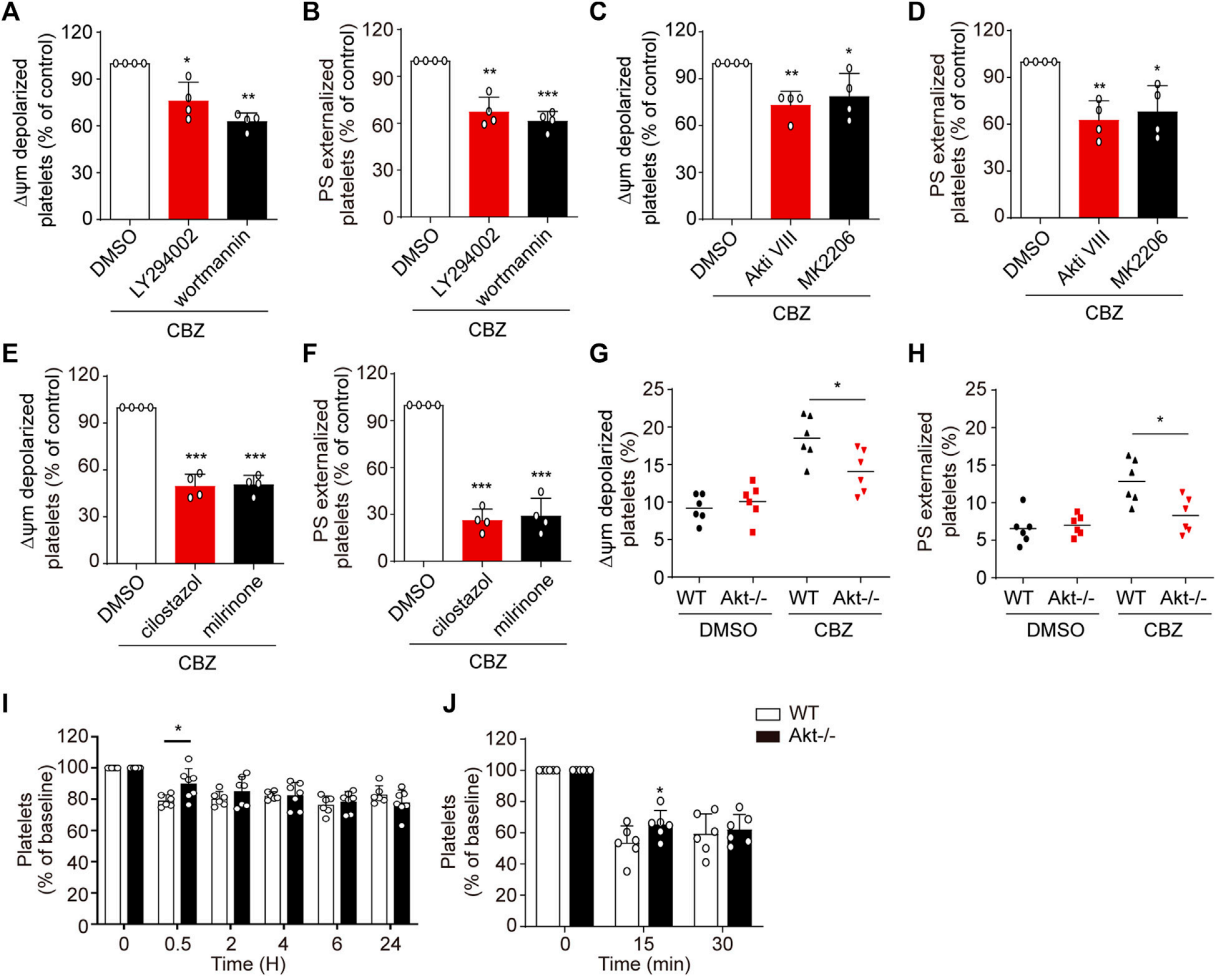
PI3K/Akt/PDE3A inhibition rescues CBZ-treated platelets from apoptosis. **(A–F)** Washed human platelets were pretreated with indicated concentrations of inhibitors at 37°C for 10 min and then incubated with 100 μM CBZ at 37°C for 70 H. **(A,B)** Flow cytometric analysis of Δψ_m_ depolarization **(A)** and PS externalization **(B)** in platelets in response to PI3K inhibitors. **(C,D)** Flow cytometric analysis of Δψ_m_ depolarization **(C)** and PS externalization **(D)** in platelets in response to Akt inhibitors. **(E,F)** Flow cytometric analysis of Δψ_m_ depolarization **(E)** and PS externalization **(F)** in platelets in response to PDE3A inhibitors. **(A–F)** Data are mean ± SD; *n* = 4; *p < 0.05, **p < 0.01, ***p < 0.001 *vs* control group, Student’s *t*-test. **(G,H)** Washed platelets from WT or *Akt* ^*−/−*^ mice were incubated with 100 μM CBZ at 37°C for 8 H Δψ_m_ depolarization **(G)** and PS exposure **(H)** were analyzed by flow cytometry. Horizontal lines indicate the median values, *n* = 6 for each group. *p < 0.05 *vs* control group by Student’s *t* test. **(I)** WT and *Akt* ^*−/−*^ mice were *i.v.* injected with a single dose of CBZ (1.2 mg/kg) and then platelet counts were assessed at the indicated time points; *n* = 6 for WT, *n* = 7 for *Akt* ^*−/−*^ mice. **(J)** C57BL/6J mice were *i.v.* transfused with calcein-labeled CBZ-treated platelets from *Akt* ^*−/−*^ or WT littermates; *n* = 6 for each group. (I and J) Data are mean ± SD; *p < 0.05, versus control group, two-way ANOVA prior to Bonferroni *post hoc* test.

CBZ mediates PKA inhibition through activation of PI3K/Akt/PDE3A signaling pathway, which leads to platelet apoptosis. Therefore, it is conceivable that PI3K/Akt/PDE3A pathway inhibition should rescue CBZ-induced platelet apoptosis. Then, we further investigated the role of PI3K/Akt/PDE3A pathway in CBZ-induced platelet apoptosis *in vitro*. Platelets were pretreated with inhibitors of PI3K/Akt/PDE3A 10 min prior to CBZ application. As shown in Figure 5 A-D that both PI3K and Akt inhibitors, which locates upstream of the signaling pathway, can only moderately reduce ΔΨ_m_ depolarization and PS exposure in CBZ-treated platelets. By contrast, PDE3A inhibitors (millirone and cilostazole) robustly reversed CBZ-induced apoptotic events (Figure 5E,F).

Since PI3K-Akt played essential roles in the upstream of PKA, we next verified the role of Akt in CBZ-induced apoptotic signaling with Akt-knockout mice. The deficiency of Akt in the *Akt* ^*−/−*^ mouse platelets was confirmed by PCR by using tail genomic DNA to amplify a 143-bp product and a 259-bp product from wild-type and Akt-knockout mice, respectively (Supplemental Figure 1). Figures 5G,H revealed that CBZ-induced apoptotic events were moderately reduced in Akt-deficient platelets, which further confirms that CBZ induces platelet apoptosis at least partly through PI3K/Akt pathway. We next assessed the effect of Akt on thrombocytopenia induced by CBZ *in vivo*. Consistent with *in vitro* data, Figures 5I,J showed that CBZ-induced thrombocytopenia and platelet clearance were slightly reduced in the *Akt* ^*−/−*^ mice. The reason may be that there is other unknown compensatory mechanism lies in the downstream of Akt signaling pathway rather than directly acting on PKA alone. These data suggest that blocking PI3K/Akt/PDE3A, especially selective PDE3A inhibition, may be a promising therapeutic strategy for CBZ induced isolated thrombocytopenia.

### CBZ Initiated Akt Activation Is Not Involved in Platelet Hyperactivity Through Simultaneous Calpain Inhibition

**FIGURE 6:**
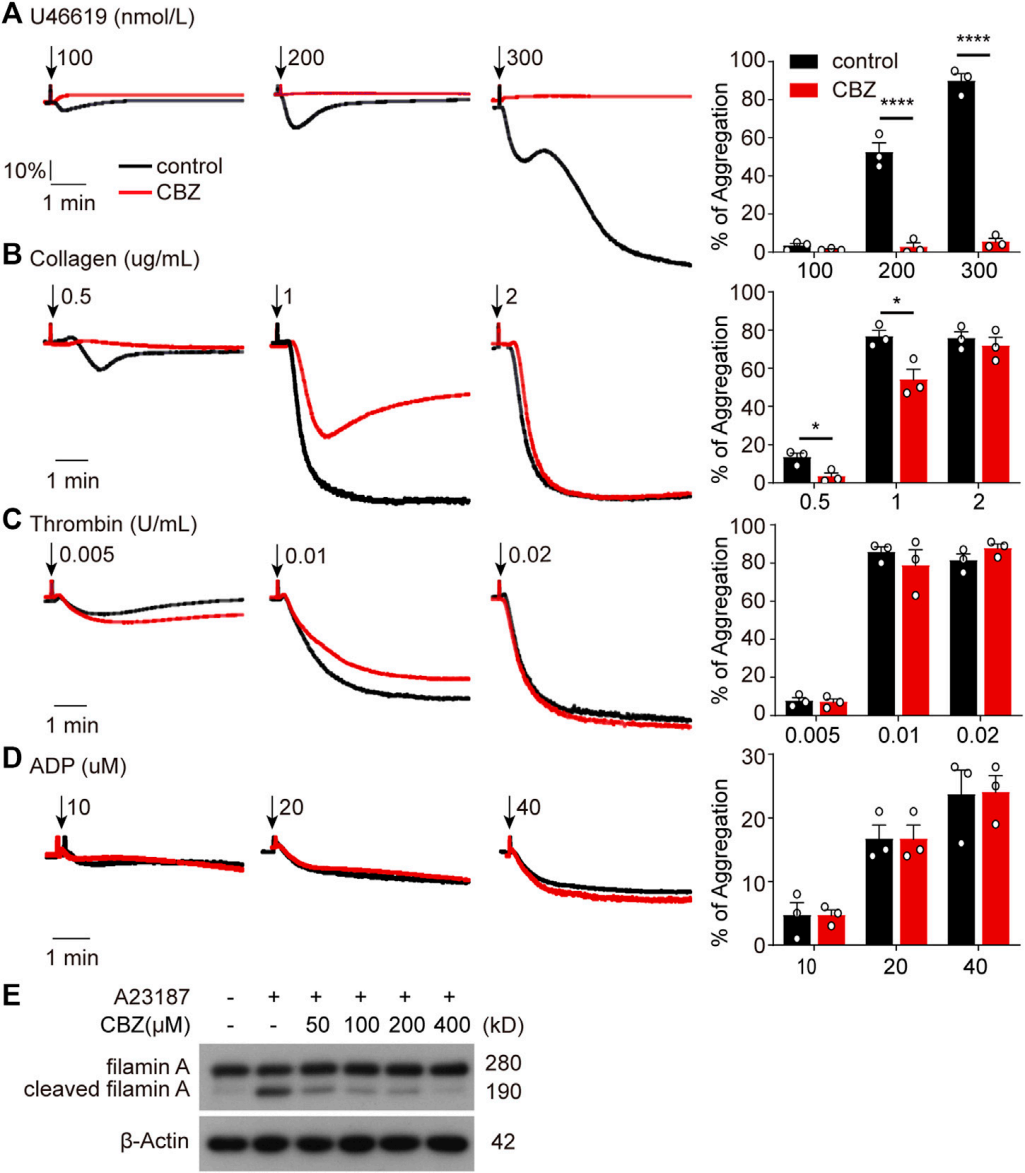
CBZ initiated Akt activation is not involved in platelet hyperactivity through simultaneous calpain inhibition. **(A–D)** Human Washed platelets **(A–C)** or PRP **(D)** were incubated with CBZ (100 μM) or vehicle (DMSO) at 37°C for 5 min. The pretreated platelets or PRP were stimulated with different concentrations of U46619 **(A)**, collagen **(B)**, thrombin **(C)**, and ADP **(D)** at 37°C under constant stirring. Platelets aggregation was monitored using a CHRONO-LOG aggregometer. Histograms of maximal platelets aggregation under the indicated conditions are shown as mean ± SD of three independent experiments; *n* = 3. *p < 0.05, ****p < 0.0001 versus control group, two-way ANOVA prior to Bonferroni *post hoc* test. **(E)** Washed human platelets were incubated with indicated concentrations of CBZ or vehicle control at 37°C for 30 min followed by 1 μM A23187 treatment at RT for 5 min. Total and cleaved filamin A were analyzed by Western blot analysis with anti-filamin A antibody.

As is shown in Figure 4 and Figure 5, platelet undergoes apoptosis through Akt activation induced by CBZ. In addition to apoptosis, Akt plays an essential role in platelet activation (Ma et al., 2019; Cheng et al., 2017; O'Brien et al., 2012). To evaluate whether CBZ treatment also incurs platelet activation, we examined platelet activity by detecting P-selectin and activated GPⅡb/Ⅲa expression on platelet membrane by flow cytometry. We found comparable levels of P-selectin and activated GPⅡb/Ⅲa (Supplemental Figure 2) among platelets stimulated with different concentrations of CBZ in resting state. As known, platelet hyperactivity may aggravate the damage of platelets, which leads to thrombocytopenia (Tang et al., 2011; Chen et al., 2008). To exclude the possibility that CBZ potentiates platelet reactivity, platelet response to several agonists stimulation was examined *ex vivo*. Surprisingly, CBZ does not aggravate, but almost completely inhibits U46619 induced platelet aggregation even to the baseline (Figure 6A). Moreover, CBZ treatment also leads to a moderate decline in platelet aggregation induced by low doses of collagen (Figure 6B). However, platelet aggregation stimulated by thrombin (Figure 6C) and ADP (Figure 6D) was not obviously reduced in CBZ treatment platelets. These data demonstrate that the isolated thrombocytopenia induced by CBZ is independent on platelet activation and hyperactivation. Also, these findings are consistent with our previous reports that apoptosis and activation occur independently downstream of Akt (Chen et al., 2018).

It has been reported that CBZ suppresses calcium overloading and calpain activation after ischemia/reperfusion (Kim et al., 2013). It is well accepted that enhanced calpain activity may account for platelet hyperreactivity (Randriamboavonjy et al., 2012). To assess the ability of CBZ in calpain inhibition, we utilized A23187, a well known calcium ionophore (Menche et al., 1980), to selectively increase the intracellular cytosolic Ca^2+^. Evaluation of the cleavage of calpain substrate filamin-A before and after CBZ treatment revealed that cleaved filamin-A fragments decreased to almost baseline levels following higher concentration application of CBZ (Figure 6G). Moreover, CBZ induces robust calpain suppression even at a lower concentration. These data indicate that Akt-dependent platelet activation is abolished through calpain inhibition in CBZ-treated platelets. At the same time, it also rules out the possibility of pathological damage caused by platelet activation during CBZ administration.

### Upregulation Murine Platelet PKA Attenuates CBZ-Induced Thrombocytopenia

**FIGURE 7:**
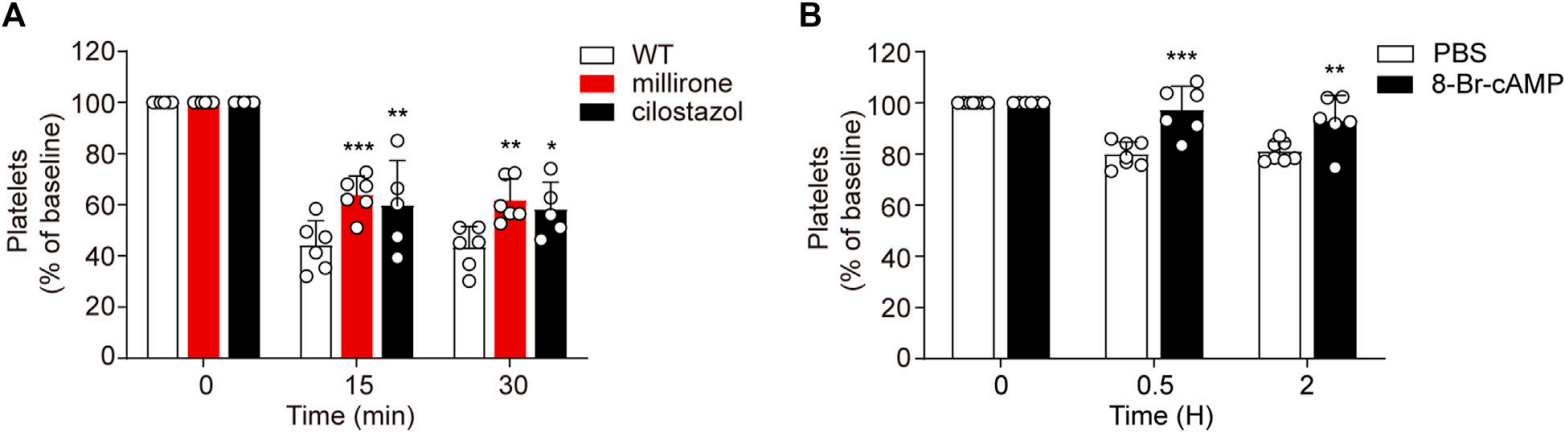
Upregulated PKA attenuates CBZ-induced thrombocytopenia. **(A)** Calcein-labeled mouse platelets pretreated with 10 μM milrinone, 10 μM cilostazol, or vehicle control were incubated with 100 μM CBZ at 37°C for 2 H and then were transfused to C57BL/6J mice. The percentage of calcein-positive platelets remaining in circulation was assessed at the indicated time points by flow cytometry; *n* = 6 for WT and milrinone group, and *n* = 5 for cilostazol group. Baseline is defined as the percentage of calcein-positive platelets immediately after transfusion. **(B)** C57BL/6J mice were injected with 2.5 mg/kg 8-Br-cAMP or PBS through the tail vein. After 30 min, 1.2 mg/kg CBZ was injected into the mice. Platelet counts were determined at the indicated time points. Platelet counts before injection is defined as the baseline; *n* = 7 for control, *n* = 6 for 8-Br-cAMP treatment group. Data are mean ± SD; *p < 0.05, **p < 0.01, ***p < 0.001 *vs* control group, two-way ANOVA prior to Bonferroni *post hoc* test.

While PI3K-Akt blockage may moderately recue platelets from apoptosis *in vitro*, PDE3A inhibitors significantly reverse platelets apoptosis in response to CBZ. Therefore, it is conceivable that upregulation of PKA activity through PDE3A inhibition may be an effective therapeutic strategy in CBZ-induced platelet clearance *in vivo*. To verify this possibility, we detected the influence of PDE3A inhibitors (milrinone and cilostazol) on CBZ induced platelet clearance with murine platelet transfusion experiment. Consist with the findings *in vitro*, milrinone and cilostazol markedly rescued CBZ-induced platelet clearance (Figure 7A).

As CBZ induced platelets apoptosis is dependent on PKA inhibition, PKA activator may prevent platelet from clearance in the context of CBZ treatment *in vivo*. Since the solvent of forskolin is DMSO, considering its toxicity *in vivo*, we employed another cAMP analog -8-bromoadenosine3’,5’-cyclicmonophosphate (8-Br-cAMP), which is known to preferentially activate PKA (Meyer and Miller, 1974), was injected into mice and then the mice were injected with 1.2 mg/kg CBZ through the tail vein. As shown in Figure 7B, 8-Br-cAMP, but not vehicle, rescues CBZ induced thrombocytopenia. Taken together, these data validate that the separate thrombocytopenia induced by CBZ is through PKA inhibition. More importantly, our results suggest that improving PKA activity is a novel therapeutic strategy for thrombocytopenia in subjects taking CBZ.

## Discussion

The involvement of long-term AEDs therapy associated fatal adverse drug reactions has drawn increasing attention. CBZ, the first generation of FDA approved AEDs, is still widely regarded as the first-line drug for epilepsy treatment, although newer AEDs has been developed. Available evidence suggests that CBZ has been considered as a definite thrombocytopenia-inducing agent, although it seems to be a rare phenomenon (Finsterer et al., 2001; Fricke-Galindo et al., 2018). Therefore, it is very important to explore the mechanism of CBZ-induced thrombocytopenia and find out possible strategies. Our results showed that CBZ dose-dependently induces platelet apoptosis *in vitro*. At present, due to its slow rate absorption, the intravenous CBZ administration is recommended for better management therapy in epilepsy patients (Marino et al., 2012). And it is accepted that intravenous AEDs is essential in the treatment of clinical seizure emergencies as well as in replacement therapy when oral administration is not possible (Conway et al., 2009; Lee et al., 2015; Patel et al., 2015; Tolbert et al., 2015). Thus, CBZ intravenous injection was employed in our experimental setup *in vivo*, and we found that platelet count decreased from 0.5 h and returned to normal level at about 6 h after injection (Figure 1). However, it is noteworthy that it takes longer time for CBZ to initiate apoptosis *in vitro*. The reason may be the fact that in addition to apoptosis, shear stress of blood flow contributes to accelerate the destruction and clearance of senile platelets. Transient thrombocytopenia caused by a single administration may recover in a short time due to a powerful platelet stock *in vivo*.

Here, we demonstrate that CBZ inhibits PKA activity, leading to platelet apoptosis *in vitro* and platelet clearance *in vivo*. This is consistent with our previous observation that PKA determines platelet life span and survival by regulating apoptosis (Zhao et al., 2017). In support of this viewpoint, forskolin, the PKA activator, and Bad knockout mice were employed to confirm the role of PKA inhibition in CBZ-induced platelet apoptosis. We found that PKA activation or genetic ablation of the PKA substrates Bad rescues CBZ-induced apoptosis obviously. Moreover, Bad deletion obviously alleviates platelet apoptosis dependent thrombocytopenia *in vivo*. This is different from some hypothesis that immune mechanism is responsible for CBZ-induced thrombocytopenia (Hoigné, 1982; Ishikita et al., 1999).

Next, we sought to identify the molecular mechanism underlying thrombocytopenia induced by CBZ treatment. CBZ has been reported to activate PI3K/Akt signaling pathway to participate in neuronal differentiation and liver regeneration (Elsherbiny et al., 2019). And we recently reported that anti-GPIbα antibody activates Akt, which elicits platelet apoptosis through activation of PDE3A and PDE3A-mediated PKA inhibition (Chen et al., 2018). Therefore, the effect of PI3K/Akt pathway on CBZ-induced PKA inhibition and platelet apoptosis needs to be further evaluated.

The influence of PI3K/Akt pathway on PKA inhibition incubated with CBZ described in this study is consistent with previous reports in which we showed that CBZ activates Akt leading to activation of PDE3A and PKA inhibition. Astonishingly, however, the inhibitors of both PI3K and Akt could only moderately rescue PKA inhibition and platelet apoptosis. In contrast, PDE3A inhibitors can significantly protect platelet from apoptosis *in vitro*. This result suggests that there may be another compensatory pathways in the upstream of PDE3A activation in platelets. Future work is needed to solve this amazing mystery.

Platelet Akt activation not only participates in platelet apoptosis, but also plays an important role in platelet activation. However, our data show that CBZ selectively provokes intrinsic programmed platelet apoptosis and rapid clearance *in vivo*. Inhibition of PI3K or Akt or genetic ablation of Akt impaired CBZ-induced apoptosis. In contrast, platelet activation is not affected by CBZ administration. It is of great significance to reduce the side effects without incurring platelet activation during the CBZ application. Platelet apoptosis and activation can occur separately downstream of Akt. It has been shown previously that calpain activation mediated by Ca^2+^ mobilization is essential for Akt-dependent platelet activation signaling (Chen et al., 2004; Bueno-Sánchez, 2020). In order to confirm the ability of CBZ in calpain inhibition, we found that CBZ markedly reduced A23187 mediated calpain activation. Therefore, our findings provide a theoretical explanation for the fact that CBZ does not lead to platelet activation, and exclude the possibility of pathological impairment incurred by platelet activation during CBZ administration.

Finally, we try to resolve the major challenge in the prevention and control of CBZ-induced isolated thrombocytopenia. We know from the aforementioned data that PI3K/Akt acts in the upstream of this signaling pathway, which not only regulates platelet apoptosis or activation, but also participates in many biological behaviors. More importantly, the efficiency of blocking PI3K/Akt in protecting platelet from apoptosis is low. Thus, it can be speculated that the blockage of PI3K/Akt signaling may be ineffective in rescuing CBZ mediated thrombocytopenia *in vivo*, and may also cause other side effects. Notably, PKA activators and PDE3A inhibitors were demonstrated to significantly reduce apoptotic events in CBZ-treated platelets (Figures 5E,F). These results implied that regulation of PKA activity may represent a strategy for extending platelet lifespan and have profound implications for the treatment of CBZ-induced isolated thrombocytopenia *in vivo*. In support of this, we verified that PDE3A inhibitors (milrinone and cilostazol) markedly reduced CBZ-induced platelet clearance. In addition, 8-Br-cAMP application can effectively prevent thrombocytopenia mediated by CBZ. Therefore, these findings suggest a profound therapeutic strategy for isolated thrombocytopenia induced by CBZ.

In conclusion, our study elucidated the molecular mechanism of CBZ-mediated selective thrombocytopenia in epileptic patients. CBZ-induced PKA inhibition incurs platelet apoptosis and accelerates rapid clearance *in vivo*. Targeting PKA appears to be a promising strategy for treating CBZ-mediated selective thrombocytopenia.

## Data Availability

The original contributions presented in the study are included in the article/Supplementary Material, further inquiries can be directed to the corresponding author/s.
