# Supplementary material for: Carbamazepine Induces Platelet Apoptosis and Thrombocytopenia Through Protein Kinase A
Source: Front Pharmacol. 2021 Sep 23;12:749930. doi: 10.3389/fphar.2021.749930 (PMC8513130; doi:10.3389/fphar.2021.749930)
Supplement: Supplementary file 1 [file DataSheet1.docx]

**
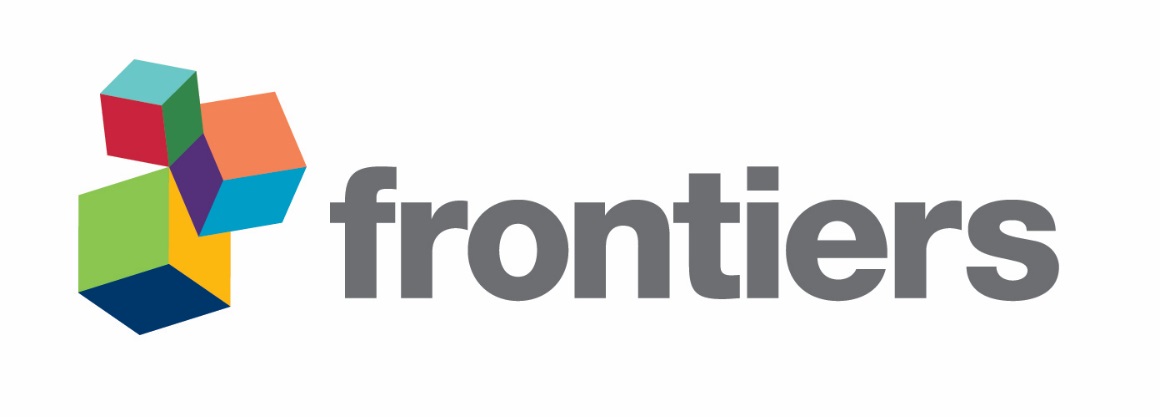
**

**Supplementary Figure 1.**

**
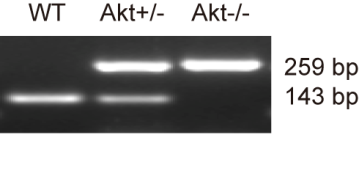
**

**Fig. S1. PCR of mouse tail DNA.** Mouse genotypes were confirmed by PCR by using tail genomic DNA to amplify a 143-bp product and a 259-bp product from WT and *Akt*^-/-^ mice, respectively. Lane 3 represents the genotype of *Akt*^-/-^ mouse.

**Supplementary Figure 2.**

**
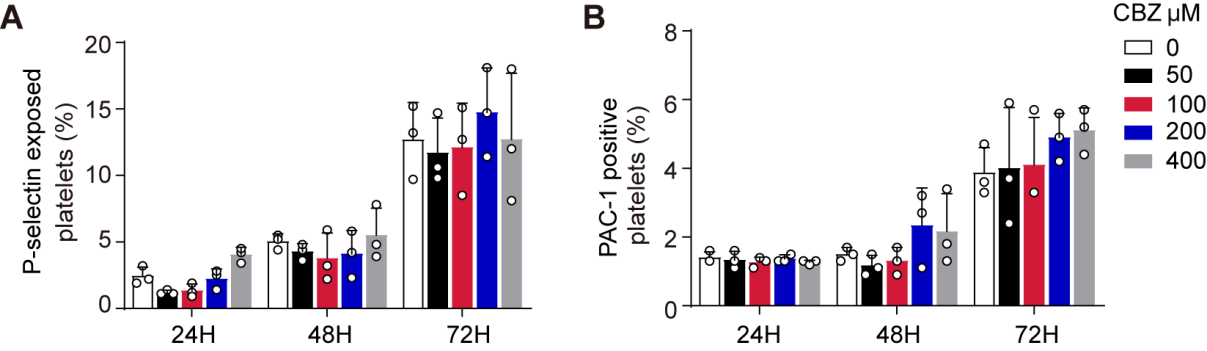
**

**Fig. S2. CBZ initiated AKT activation does not involve in platelet activation.** (A and B) Washed human platelets were incubated with indicated concentrations of CBZ or vehicle control at 37 °C for 24 H, 48 H and 72 H. Platelet P-selectin externalization (A) and PAC-1 binding (B) were determined by flow cytometry, *n*=3. Data are expressed as mean ± SD by two-way ANOVA followed by Dunnett’s *post hoc* test.
